# Cu(II) Complexes of 4-[(1*E*)-*N*-{2-[(*Z*)-Benzylidene-amino]ethyl}ethanimidoyl]benzene-1,3-diol Schiff Base: Synthesis, Spectroscopic, In-Vitro Antioxidant, Antifungal and Antibacterial Studies

**DOI:** 10.3390/molecules23071581

**Published:** 2018-06-29

**Authors:** Ikechukwu P. Ejidike

**Affiliations:** Department of Chemistry, Faculty of Applied and Computer Sciences, Vaal University of Technology, P.O. Box X021, Vanderbijlpark 1911, South Africa; destinedchild12@gmail.com or ikechukwue@vut.ac.za; Tel.: +27-84-567-5918

**Keywords:** copper(II) complexes, 2′,4′-dihydroxyacetophenone, unsymmetrical Schiff base, DPPH assay, ABTS assay, antimicrobial activity

## Abstract

The current study reports the synthesis of copper complexes of a tridentate Schiff base ligand. The compounds of the type [Cu(L)X]∙n(H_2_O) (where L = tridentate ONN Schiff base ligand, X = Cl^−^, Br^−^, SCN^−^, NO_3_^−^, CH_3_COO^−^), were characterized on the basis of elemental analyses, FT-IR, UV-vis, molar conductance, ^1^H-NMR, XRD and thermal analyses. The spectra revealed that the Schiff base ligand acts as a tridentate ligand through two azomethine nitrogen atoms and a phenolic oxygen atom. The molar conductance measurements of the complexes in DMF correspond to non-electrolytic nature. TGA and DTA studies results gave insight into the dehydration, thermal stability, and thermal decomposition. Square-planar geometry has been assigned to the prepared complexes as indicated by the electronic spectral measurements. Cu(II) compounds showed antiradical potential against DPPH and ABTS radicals. The antimicrobial potential of the Schiff base ligand and its Cu(II) complexes were evaluated by the rapid *p*-iodonitrotetrazolium chloride (INT) colorimetric assay against some selected bacteria strains: *Staphylococcus aureus* and *Enterococcus faecalis* (Gram +ve); *Klebsiella pneumoniae* and *Pseudomonas aeruginosa* (Gram −ve), and fungi (*Candida albicans* and *Cryptococcus neoformans*). The compounds showed a broad spectrum of antibacterial and antifungal activities, with MIC values ranging from 48.83 to 3125 μg/mL.

## 1. Introduction

Transition metals are associated with biological processes that are indispensable to life processes. Thus, they can coordinate with O- or N-terminals from proteins in a variety of models, and thereby, play a vital role in the conformation and utility of living macromolecules [1,2], and act as antimicrobial agents [3]. Schiff bases containing imino moieties (>C=N–) and their metal complexes have been widely reported to exhibit a variety of interesting biological activities such as antimicrobial, anti-inflammatory, antifungal, antitumor, anticancer activities, antiproliferative and analgesic effects [2,4,5,6,7]. Metal complexes containing some bioactive ligands are sometimes more effective regarding biological and pharmaceutical applications than the free ligands [8].

2-Hydroxy-1-(4-hydroxyphenyl)ethenone Schiff bases are of particular interest due to their wide range of biological activities, as shown by 4-[(1*E*)-*N*-(2-aminoethyl)ethanimidoyl]benzene-1,3-diol [9], and antioxidant and antibacterial activities exemplified by derivatives of 1-phenylbutan-1-one Schiff base [10]. Other activity includes structural activity demonstrated by octahedral and tetrahedral complexes of naphthalene-2-ol derivative [11]. Cu(II) complexes of the Schiff base (7*E*)-7-(3-ethoxy-2-hydroxybenzylideneamino)-4-methylquinolin-2(1*H*)-one were reported as a potential therapeutic agent [12]. Copper(II) complexes of the quinolin-4(3*H*)-one Schiff base ligand derived from the reaction of 3-amino-2-methyl-4(3*H*)-quinazolinone with different substituted aromatic aldehydes was reported [13] to act as avid binding and cleaving agent. There has been recent attention towards metal coordination compounds acting as antioxidants in protecting living organisms and cells from impairment associated with oxidative stress or free radicals [14]. Tridentate and tetradentate Schiff bases and their metal complexes incorporating *N*-, *O*-donors have been reported to exhibit moderate to strong scavenging activity on DPPH and ABTS radicals [9,14].

In an effort towards the development of metal-based chemotherapeutic agents with interesting biological potential such as bactericidal activity alongside an important role in protecting the body against damages caused by reactive oxygen species [9,10,14], we have described the synthesis and biological potential of the tridentate Schiff base ligand: [4-[(1E)-N-{2-[(Z)-benzylideneamino]ethyl}-ethanimidoyl]benzene-1,3-diol] (BEB) derived from the condensation of ethylenediamine, 2′,4′-dihydroxyacetophenone and benzaldehyde, using reflux methods. After the complexation with copper(II) ion, the ligand and its complexes were characterized by their elemental analyses, molar conductivity, Fourier transform infrared spectroscopy (FT-IR), ultraviolet-visible spectroscopy (UV-Vis), X-ray diffractometer (XRD), and thermogravimetric analysis (TGA). Also, the antibacterial and antifungal activities of the synthesized compounds were investigated against microscopic strains, while their antioxidant activity was measured by their ability to scavenge DPPH and ABTS* free radicals.

## 2. Results and Discussion

### 2.1. Synthesis and Characterization of the Compounds

**Figure 1:**
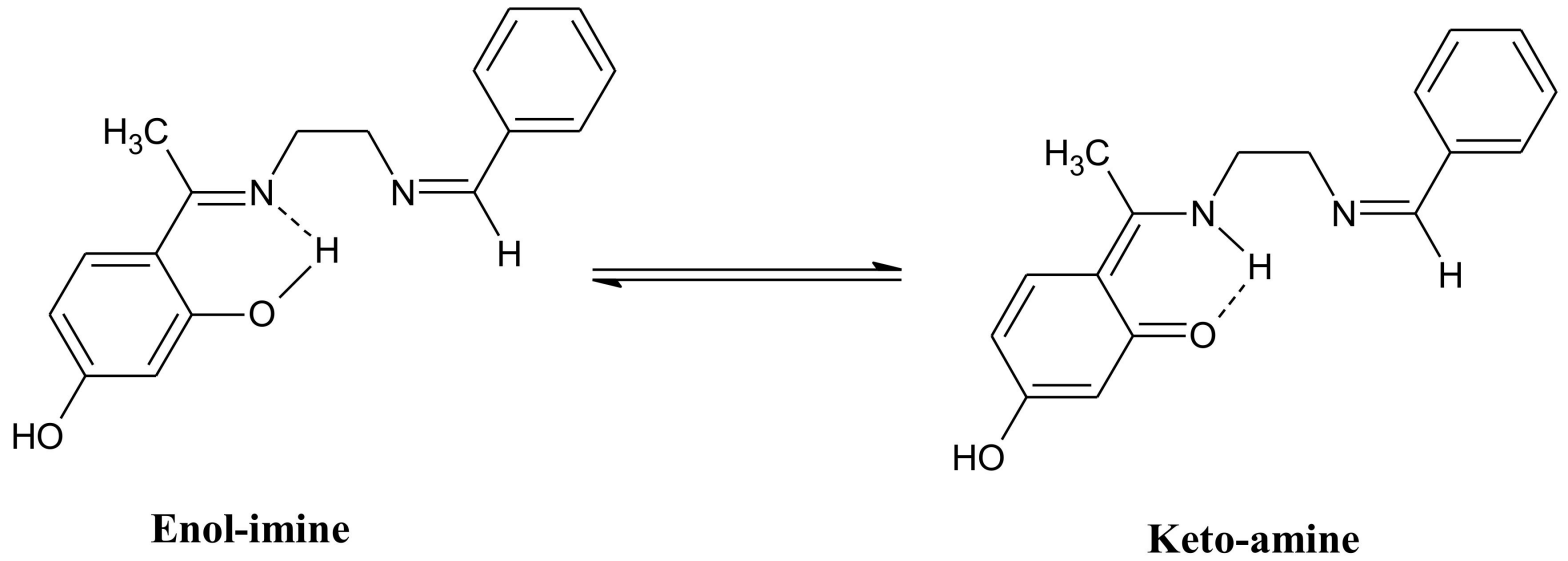
Proposed structure of the ligand (BEB).

**Scheme 1:**
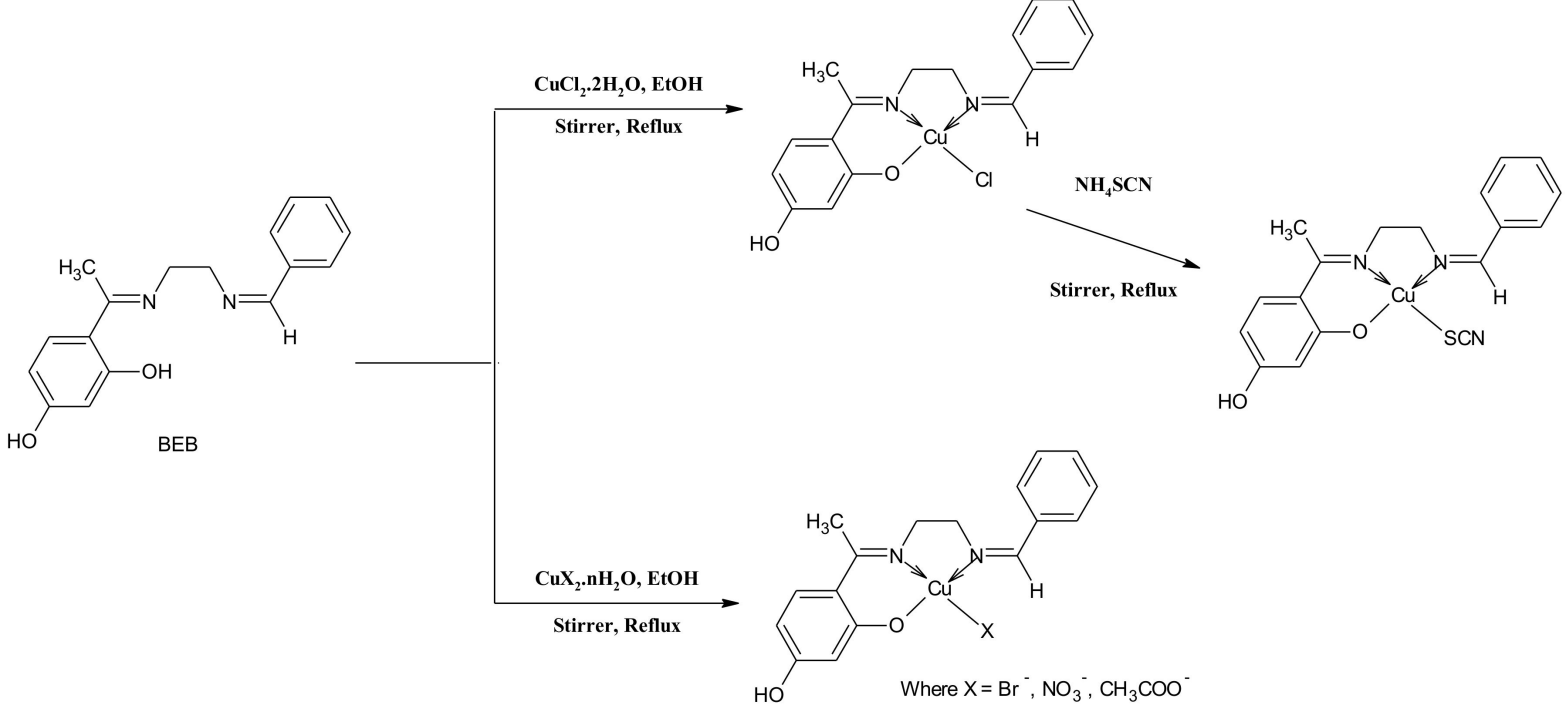
Proposed structures of the newly obtained copper(II) complexes.

The synthesized Schiff base ligand 4-[(1*E*)-*N*-{2-[(*Z*)-benzylideneamino]ethyl}ethanimidoyl]-benzene-1,3-diol (BEB) was subjected to elemental analyses, IR, UV-vis and NMR spectral studies. Elemental analyses results (C, H, and N) were in good agreement with those required by the proposed formula expressed in Table 1, Figure 1. Reaction of CuX_2_·nH_2_O with ONN Schiff base ligand (in ethanol under reflux) yielded greyish to dark-blackish coloured complexes with the general formula [Cu(L)X]∙n(H_2_O) (where L = tridentate Schiff base ligand, X = Cl^−^, Br^-^, SCN^−^, NO_3_^−^, CH_3_COO^−^) (see Scheme 1).

The ligand (BEB) displayed significant chelating properties, as showed by the immediate precipitation of the complexes when the ligand (BEB) comes in contact with Cu^2+^ salt forming complexes. The micro-analytical data (Table 1) indicate that the metal to ligand ratio is 1:1 for all the complex systems. All the copper complexes are non-hygroscopic, coloured, and air-stable at room temperature. The as-synthesized complexes show very low solubility in most organic solvents but were found soluble in dimethylformamide (DMF) and dimethyl sulfoxide (DMSO). The characteristic IR spectrum data of the ligand (BEB) showed a broad and weak band centered around 2590 cm^−1^ due to ν(O‒H) in O‒H---N intramolecular hydrogen-bonded (Figure 1) in the free ligand [15,16]. A characteristic stretching vibration band at 1613 cm^−1^ is assigned to ν(C=N) azomethine group in the BEB ligand. 

### 2.2. Molar Conductivity Measurements

The observed molar conductance (*Λ*μ) of the Cu(II) complexes (Table 1) solutions in 10^−3^ DMF, indicate the non-ionic nature of the compounds and they are considered as non-electrolytic [10,17]. The conductivity values were found in the range 10.71–22.70 μS cm^−1^. Furthermore, it indicates the bonding of the anions to the copper cation and the complexes may be formulated as [Cu(L)X] (where L = tridentate Schiff base ligand, X = Cl^−^, Br^−^, SCN^−^, NO_3_^−^, CH_3_COO^−^) respectively [18].

### 2.3. Infrared Absorption Spectra

The relevant IR data for the ligand (BEB) and its corresponding Cu(II) complexes are presented in Table 2. The infrared of BEB displayed characteristic bands at 3404 cm^−1^ that could be attributed to the ν(O‒H) stretching vibrations in the free Schiff ligand [10,16]. The absence of this band in the spectra of the complexes is an indication of deprotonation and involvement of the phenolic hydroxyl group of the ligand during bond formation with the copper ions [19,20]. The medium and broadband at 3378–3396 cm^−1^ in the spectra of the Schiff base-Cu(II) complexes are assigned to the ν(OH) frequency of the water molecules in the complexes [21]. This was further supported by the downward shift in the phenolic ν(C−O) group observed at 1266 cm^−1^ in the free ligand to the region 1237–1246 cm^−1^ [22] upon complexation. This shift confirms the coordination of the phenolate oxygen to the metal ion [14,20].

The strong band observed at 1613 cm^−1^ in the spectra of the free Schiff base ligand (BEB) is a characteristic of the ν(C=N) azomethine stretching vibration [10,16,19]. This vibration underwent a shift to lower frequency 1588–1590 cm^−1^ upon complexation, indicating the bonding of nitrogen of the azomethine group to the Cu ions [11,12,15]. The donation of electrons from nitrogen to the empty d-orbitals of the Cu(II) ions further explain this downward shifting of the ν(C=N) moiety [23,24]. [Cu(BEB)SCN] complex show a unique and prominent vibrational band between 2161 and 2096 cm^−1^ assignable to ν(N=C=S) vibrational stretching of the thiocyanate group [25]. 

Copper complex of the nitrato group showed two vibrations around 1417 and 1297 cm^−1^, absent in the tridentate Schiff base (BEB). The stretching vibrations separated by 120 cm^−1^ suggest the unicoordination pattern of nitrate ion within the complex sphere [11]. The asymmetric carboxyl ν_asym_(COO^−^) in [Cu(BEB)CH_3_COO] shifted to a higher frequency 1538 cm^−1^ region while the shift to a lower frequency region 1340 cm^−1^ is assigned to symmetric carboxyl ν_sym_(COO^−^), indicating linkage of the carboxylate oxygen atom to the Cu(II) ion. The difference between these vibrational bands showed a separation value of 198 cm^−1^, this suggests the monodentate binding of carboxylate group to Cu(II) ion [11,25,26]. The ring skeletal vibrations ν(C=C) were consistent in both the ligand (BEB), the Cu(II)-Schiff base complexes and were unaffected by complexation [16]. Furthermore, the appearance of new bands between 526 and 597 cm^−1^ are attributed to ν(Cu‒N); while the bands within the region of 452–462 cm^−1^ are assigned to ν(Cu‒O) [15,21,24]. The FTIR spectra data confirmed that the Schiff base (BEB) behaves as a tridentate ligand coordinated to the Cu(II) ions via the two groups of the imino nitrogen and one phenolic oxygen atom.

### 2.4. ^1^H-NMR Spectral Studies

The ^1^H-NMR spectrum of the Schiff base ligand (BEB) was recorded in DMSO-*d*_6_ at room temperature using TMS as an internal standard. It is significant to highlight that the proton resonance of the O–H group at δ 9.76 ppm is due to the presence of intramolecular hydrogen bonding [18,26,27]. The single proton resonances in the ^1^H-NMR spectra of the ligand occurring at δ 8.39 ppm have been assigned to the azomethine group proton [16,21]. In the spectrum, phenyl (benzaldehyde) protons (m, 5H, C_6_H_5_) was observed at δ 7.25–7.74 ppm. The protons at the 5 and 6 positions of the 2′,4′-dihydroxyacetophenone ring come up as multiplets at δ 6.04–6.14 ppm (m, 2H, –C_2_H_2_), while those at position 3 occur as a singlet at δ 6.36 ppm (s, H, –CH), and the enolic OH appears at δ 5.39 ppm (s, 1H). The aliphatic protons (s, 4H, –C_2_H_4_) in the ethylenediamine chain show a singlet peak at δ 3.83 ppm. The peak at δ 2.09 ppm (s, 3H, N=C–CH_3_) is due to methyl protons of the azomethine group in the Schiff base ligand [15,16,18,19,26].

### 2.5. Electronic Spectral Measurements

The electronic spectral data of the Schiff base (BEB) and its Cu(II) complexes recorded in DMF solution at room temperature at a wavelength range of 200–800 nm is listed in Table 3. The spectra of the free ligand exhibit three strong peaks at 277, 309 and 381 nm. The two bands at 277 and 309 nm are attributed to π-π* transitions of the enol-imine tautomer of the Schiff base [10,26]. The third band (hypsochromic shift band) in the spectra of the Schiff base (381 nm) is assigned to n-π* transition involving molecular orbitals of the (>C=N) azomethine groups. All the Cu(II) complexes show two important absorption bands which are similar to the absorption spectra of the Schiff base (BEB), this bands shifted to lower and/or higher wavelength in the regions 335–325 nm, π-π* transitions for the copper complexes respectively, Table 3 [10,15,18].

**Figure 2:**
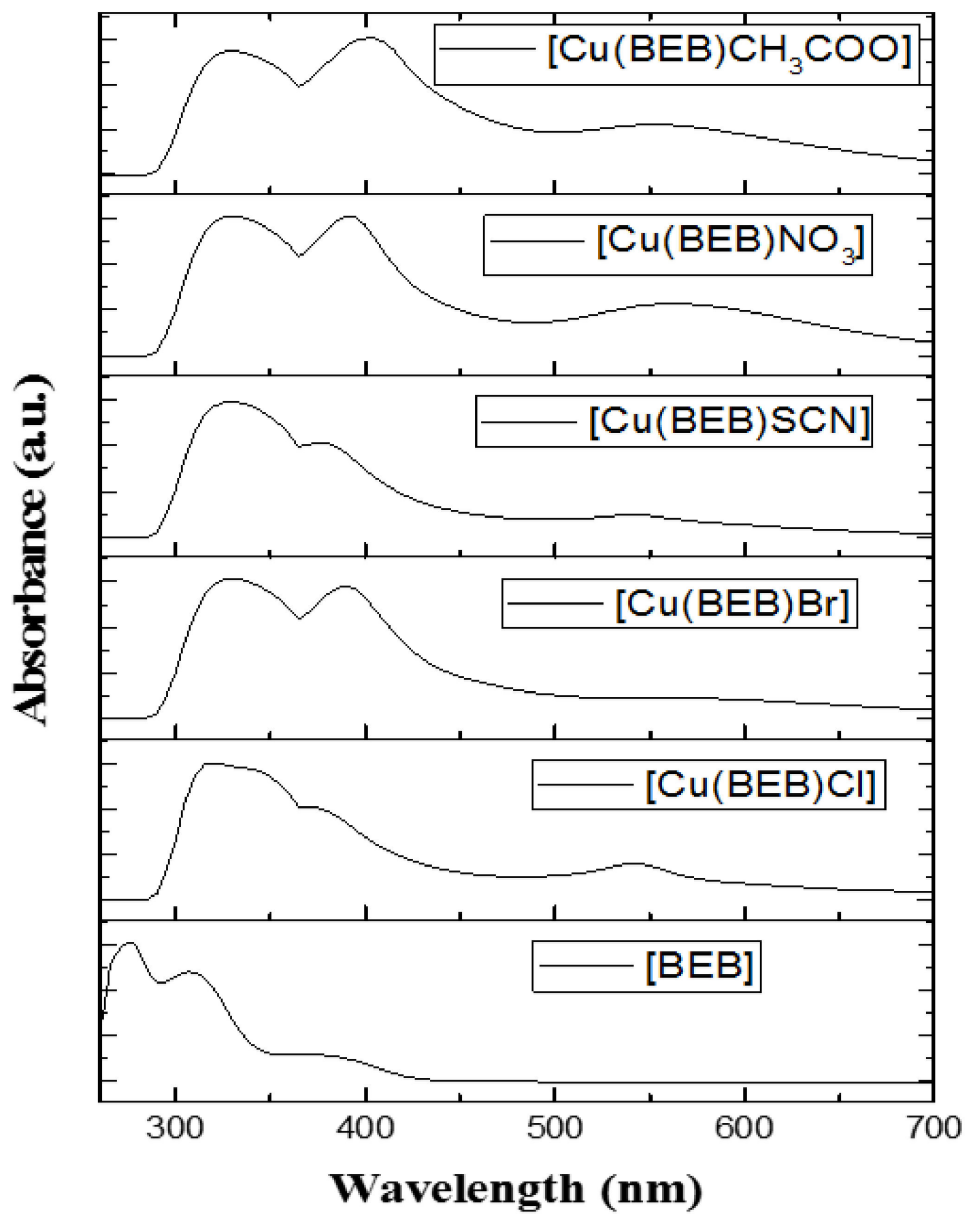
Electronic visible absorption spectra of Cu(II) complexes of Schiff base (BEB) in DMF.

The band in the ligand around 381 nm (n-π* transition) shifted to a lower and higher wavelength 405–375 nm (Figure 2). This shift may be ascribed to the donation of lone pairs of nitrogen atoms of the azomethine group of the Schiff base to the Cu(II) ions (M ← N) LMCT [16,23]. Electronic spectra of the copper(II) complexes display one absorption of a broad low-intensity band in the regions 570–545 nm. This band is attributed to the d-d transitions of the copper(II) ions and its assignable to ^2^B_1g_ → ^2^A_1g_ (d*x*^2^ − *y*^2^ → d*z*^2^). Square-planar geometry is therefore confirmed for the as-synthesized complexes [10,16,26]. The order of *d-d* transition of the Cu(II) ions with respect to their corresponding anions (Figure 2) can arranged in the order: Cl^−^ < SCN^−^ < Br^−^ < CH_3_COO^−^ < NO_3_^−^, signifying the modification on the electronic spectra of the copper complexes is dependent on the type of ligand (anions coordinated).

### 2.6. Powder X-ray Diffraction Spectroscopy

**Figure 3:**
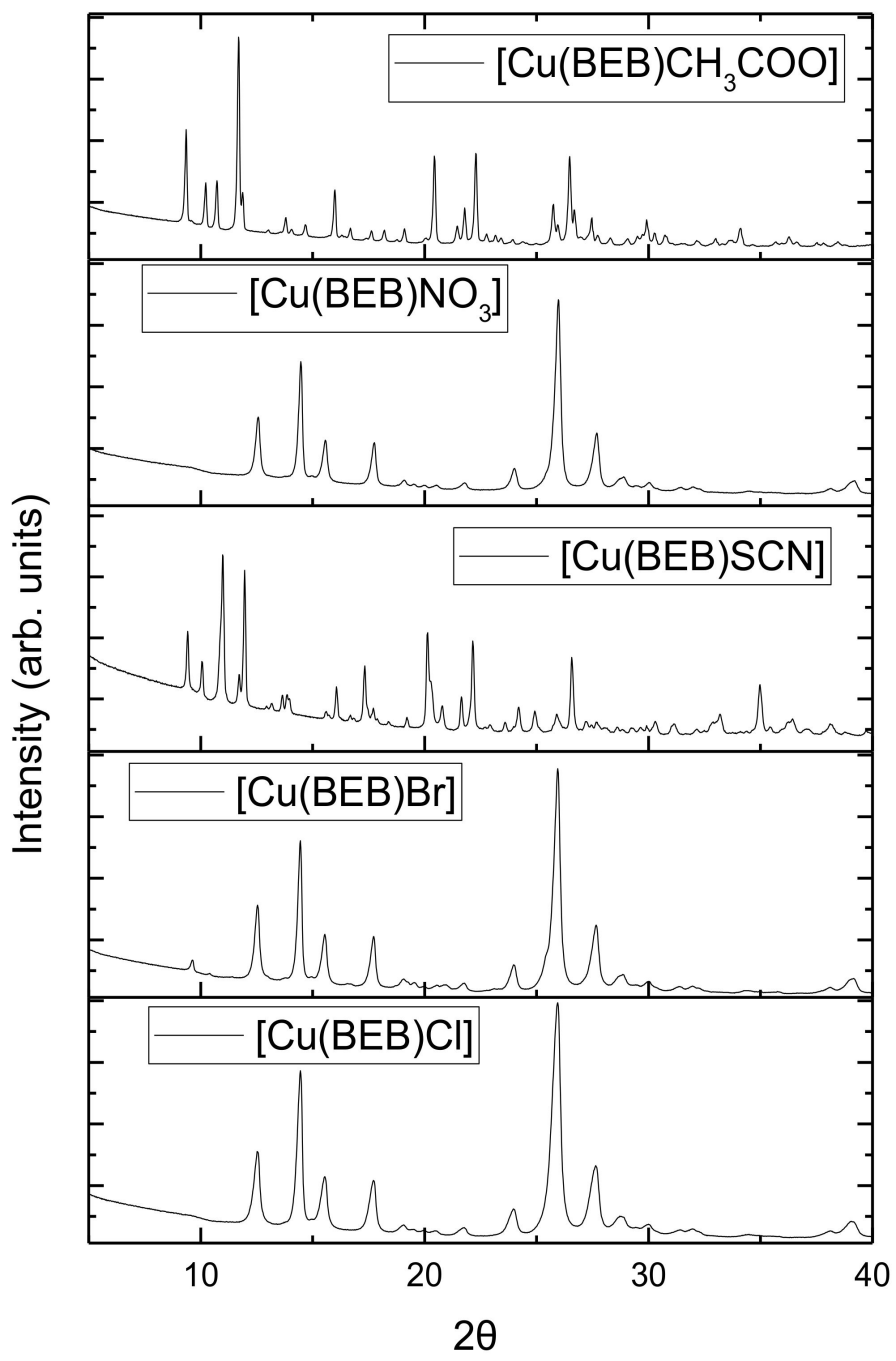
Powder XRD pattern of the Cu(II) Schiff base complexes.

XRD pattern of the Cu(II) complexes bearing Cl^−^, Br^−^, SCN^−^, NO_3_^−^, CH_3_COO^−^ were recorded in the range (2θ = 0–60) is shown in Figure 3. The pattern of the metal complexes was studied to further obtain evidence about the structure of the metal complexes at wavelength 1.5406 Ǻ. The XRD pattern of the Cu(II) complexes shows well defined crystalline peaks indicating that the Cu(II)-Schiff base complexes were in crystalline phase [28]. The average crystallite size of the complexes *d*_XRD_ was estimated from XRD patterns by Scherer’s formula [29]:

*d*_XRD_ = 0.9λ/β (cosθ)

where ‘λ’ is the wavelength, ‘β’ is the full width at half maxima and ‘θ’ is the diffraction angle. The XRD reveal that [Cu(BEB)Cl], [Cu(BEB)Br], [Cu(BEB)SCN], [Cu(BEB)NO_3_] and [Cu(BEB)CH_3_COO] complexes have the average crystallite sizes of 27, 31, 56, 32, 38 nm respectively. It suggests the complexes are nanocrystalline [23,29].

### 2.7. Thermal Decomposition of the Complexes

**Figure 4:**
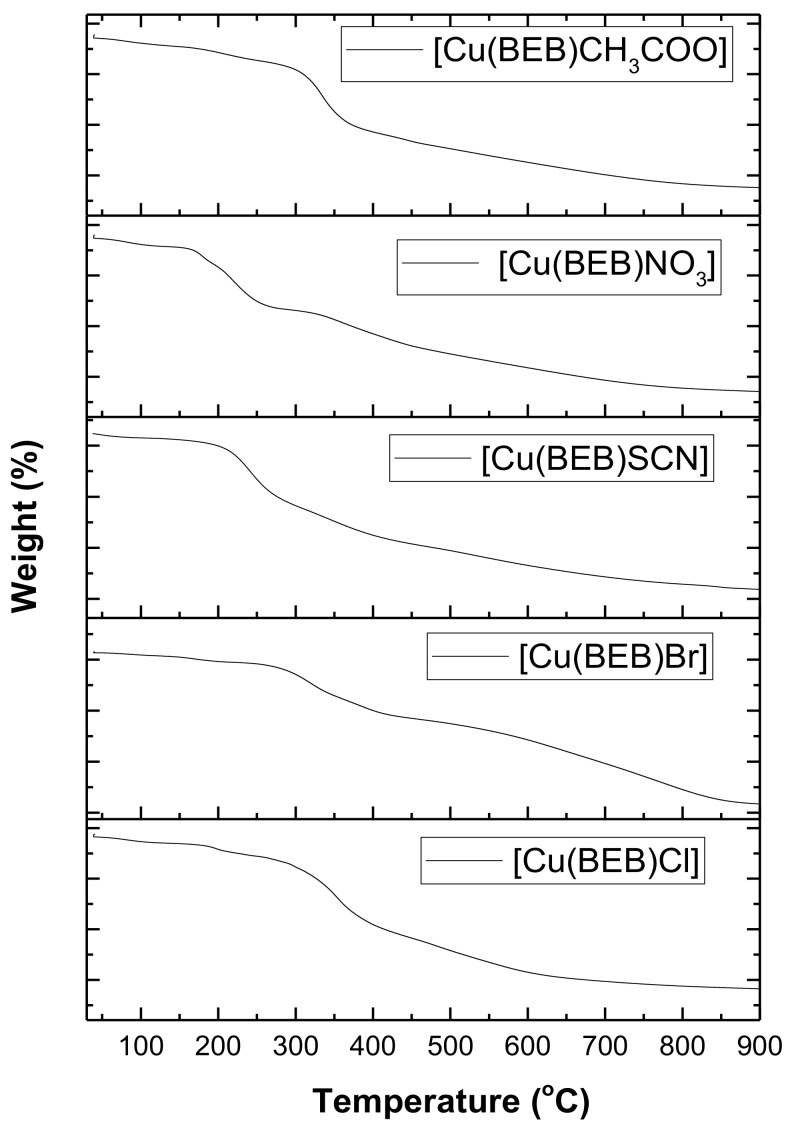
TG curves of the Cu(II) Schiff base complexes.

The thermal degradation of Cu(II) complexes bearing different anions was studied using thermogravimetric techniques. Thermogravimetric and derivative thermogravimetric analysis (TGA/DTA) of the synthesized Schiff base compounds were measured under a nitrogen atmosphere at a heating rate of 10 °C min^−1^ from 20 to 900 °C. TG/DTG results were plotted as percentage weight loss against temperature; provides insight into nature, properties of various molecules and the residues obtained after thermal decomposition [21,23,29]. Decomposition of the complexes takes place in several steps. Water molecules lost between 45 and 150 °C, gave rise to metal oxides above 450 °C for the Cu(II) complexes (Figure 4).

[Cu(BEB)Cl]·2H_2_O, was thermally decomposed in four successive decomposition steps. The decomposition steps were in the temperature ranges 46–121 °C, 149–250 °C, 286–431 °C, 455–543 °C. The DTG curve gives an exothermic peak at 83 °C, 196 °C, 355 °C, 481 °C (the maximum peak temperature), caused by dehydration step; indicating the presence of water molecule in the complex, loss of chlorine, breakdown of the ligand and Schiff ligand molecule and the formation of CuO. The overall estimated weight loss amounts to 38.3% (calc. 41.4%).

The decomposition of the [Cu(BEB)Br]·H_2_O, takes place in four decomposition steps within the temperature range 54–841 °C. The decomposition steps were in the temperature ranges 54–110 °C, 135–220 °C, 222–446 °C, 547–841 °C, with DTG curves with exothermic peaks at 85 °C, 167 °C, 320 °C, 643 °C (the maximum peak temperature) respectively; caused by dehydration step indicating the presence of water molecule in the complex, loss of bromine, breakdown of the ligand and Schiff ligand molecule and the remaining mass seems likely to correspond to CuO as a residue (Figure 4). The overall estimated weight loss amounts to 62.2% (calc. 68.4%).

[Cu(BEB)SCN]·2H_2_O was stable up to 43 °C, and its decomposition started at 45 °C and finally completed at 453 °C. In the DTA thermogram of the [Cu(BEB)SCN]·2H_2_O, it reveals three exothermal peaks at 58 °C, 242 °C, 340 °C (the maximum peak temperature); caused by loss of water molecule, thiocyanate and ligand; and Schiff ligand molecule, the remaining mass seems likely to correspond to CuO. The overall estimated weight loss amounts to 32.9% (calc. 34.4%).

[Cu(BEB)NO_3_]·3H_2_O was stable up to 46 °C and its decomposition started at 49 °C and finally completed at 452 °C. In the DTA thermogram of the [Cu(BEB)NO_3_]·3H_2_O, it reveals four exothermal peaks at 89 °C, 181 °C, 216 °C, 363 °C (the maximum peak temperature); and TG temperatures of 49–120 °C, 148–193 °C, 193–286 °C, 315–452 °C caused by loss of water molecule, nitrate, breakdown of the ligand and Schiff ligand molecule, the remaining mass seems likely to correspond to CuO. The overall estimated weight loss amounts to 35.1% (calc. 36.5%).

The decomposition of the [Cu(BEB)CH_3_COO]·2H_2_O complex takes place in four decomposition steps within the temperature range 50–472 °C. The decomposition steps were in the temperature ranges 50–146 °C, 146–254 °C, 263–392 °C, 425–472 °C. The DTG curve gives an exothermic peak at 77 °C, 207 °C, 336 °C, 446 °C (the maximum peak temperature); caused by dehydration step indicating the presence of water molecule in the complex, loss of acetate, breakdown of the ligand and Schiff ligand molecule and the formation of CuO. The overall estimated weight loss amounts to 34% (calc. 36%).

Additionally, it has been shown that the electronegativity and the atomic radius of the central metal atom also affect the thermal stability bonding [26,27]. Considering the loss of organic moiety as the decomposition temperature, the thermal stability of the copper(II) complexes can be represented with respect to anions as Br^−^ > Cl^−^ > CH_3_COO^−^ > NO_3_^−^ > SCN^−^.

### 2.8. Antioxidant Assays

The oxidative damages caused by reactive oxygen species (ROS) on lipids, proteins, and nucleic acids may generate various chronic diseases, such as coronary heart disease, atherosclerosis, cancer, and aging [2,24]. It is significant to administer chemotherapeutic drugs that may be rich in antioxidants, to avert free radical impairment in the body. The capability of Schiff bases and their metal complexes to scavenge free radicals is a significant property. In this study, the results of DPPH and ABTS scavenging ability of the Schiff base (BEB) and Cu(II) complexes in DMF as solvent are presented. The antioxidant assay was carried out using different concentrations of the test samples, while Ascorbic acid (vitamin C), rutin, gallic acid and butylated hydroxytoluene (BHT) were employed as standard agents. 

#### 2.8.1. DPPH Radical Scavenging Assay

**Figure 5:**
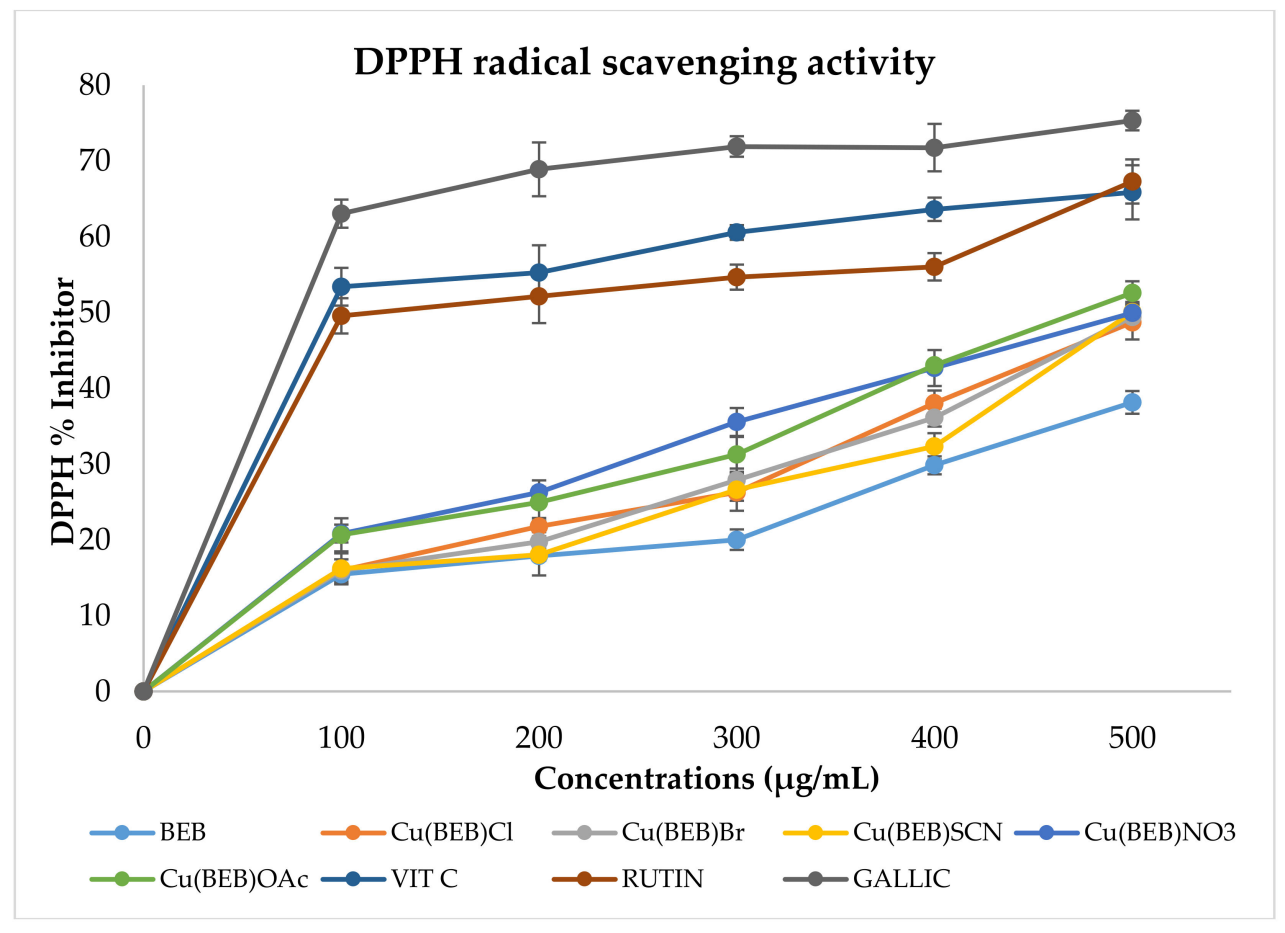
DPPH free radical scavenging assays of BEB and its Cu(II) complexes (n = 3).

The quantitative antioxidant activity of Schiff base (BEB) and its Cu(II) complexes were determined spectrophotometrically by the DPPH assay alongside the standards rutin, gallic acid and ascorbic acid, as displayed in Figure 5. The observed DPPH activities of the tested sample possess strong proton donating power as compared to those of the standards. Reduction in the DPPH radical abilities was calculated based on the decrease in its absorbance at 517 nm prompted by antioxidants [10,16]. The chelated Cu(II)-Schiff base complexes exhibited significant DPPH radical scavenging ability in all the concentrations studied than the corresponding free ligand (BEB). IC_50_ and its corresponding R^2^ (correlation coefficient) values of tested compounds are listed in Table 4.

However, the Cu(II) complexes showed comparable or lower scavenging activity as compared to the standard agents with [Cu(BEB)NO_3_] showing significantly higher scavenging ability (IC_50_ = 2.01 ± 1.49 µM) than rutin (2.49 ± 1.27 µM). At the highest concentration (500 μg/mL) the activity of the free ligand (BEB) was observed to be 38.1%. However, upon copper complexation, it increased significantly within the range 48.72–52.5% from [Cu(BEB)Cl] to [Cu(BEB)CH_3_COO] (Figure 3). The observed discolouration further supported it from purple DPPH radical solution to yellow solution showing scavenging of the DPPH radicals by hydrogen donation. Scavenging ability of the test samples on the DPPH radical can be ranked in the following order: [gallic acid] > [Vit. C] > [Cu(BEB)NO_3_] > [rutin] > [Cu(BEB)SCN] > [Cu(BEB)CH_3_COO] > [Cu(BEB)Br] > [Cu(BEB)Cl] > [BEB]. Therefore, these compounds could be used as promising therapeutic agents for developing therapeutic agents for treating stress-induced pathological conditions [10].

#### 2.8.2. 2,2′-Azino-bis(3-ethylbenzothiazoline-6-sulfonic Acid) Radical Scavenging Activity

**Figure 6:**
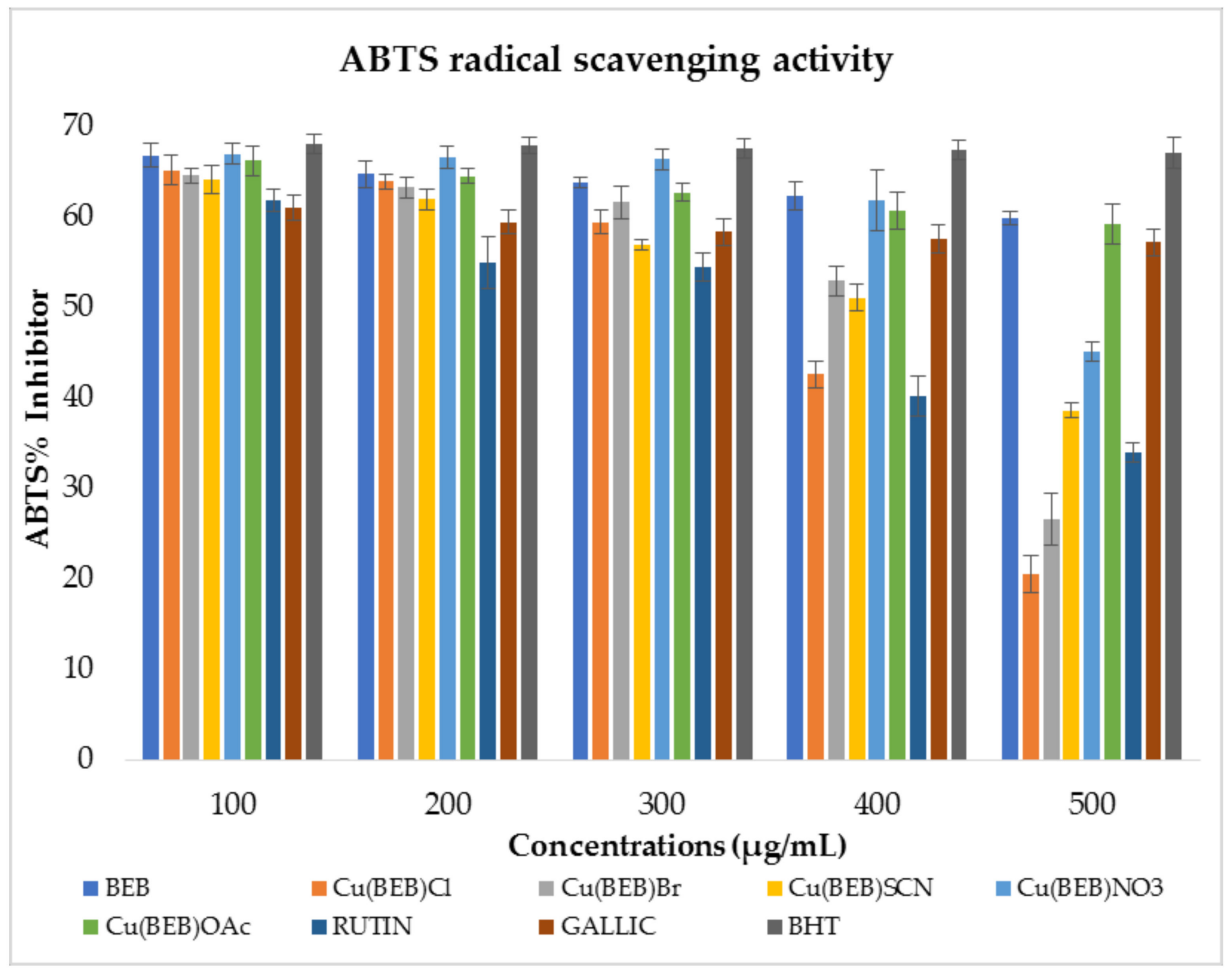
ABTS activities of BEB, its Cu(II) complexes and standard drugs (n = 3).

The Schiff base and its complexes were further screened for free radical scavenging activity by the ABTS method [14]. The percentage inhibition results of free radical scavenging activity of the test samples are shown in Figure 6. At 734 nm, the absorbance of the active ABTS^+^ solution [14,16] declined upon the addition of different concentrations of heterocyclic Cu(II) complexes, and the same trend was also observed for the standard drugs with the percentage ABTS inhibition [24]. The assay measures radical scavenging by electron donation. The coordination of metal ions to the Schiff base (BEB) resulted in a lower spectrum of activity comparable to that of the ligand (2.77 ± 1.44 µM) and standards for drugs used [rutin (2.86 ± 0.92 µM), gallic acid (1.22 ± 1.08 µM) and BHT (2.31 ± 1.30 µM)]. Lowest concentrations of the test samples were effective in quenching ABTS^+^ radicals in the system. The scavenging of the ABTS^+^ radical by the Schiff base and its metal complexes was found to possess moderate to high activities [14,16,24].

[Cu(BEB)Cl] complex exhibited the highest ABTS scavenging activity amongst the studied copper(II) complexes, with an IC_50_ value of 3.02 ± 0.47 µM and 0.852 R^2^ (correlation coefficient) while [Cu(BEB)NO_3_], exhibited the least activity amongst the complexes with an IC_50_ value of 4.56 ± 1.99 µM (Table 4). The ABTS scavenging activity pattern of the test compounds can be ranked in the following order: [gallic acid] > [BHT] > [BEB] > [rutin] > [Cu(BEB)Cl] > [Cu(BEB)SCN] > [Cu(BEB)Br] > [Cu(BEB)CH_3_COO] > [Cu(BEB)NO_3_]. This result shows that the compounds in this study possess the ability to scavenge different free radicals in different systems, indicating that they may be useful as therapeutic agents for treating pathological damage associated with the radical generation and for averting cell oxidative damage [16,24].

### 2.9. In Vitro Antimicrobial Activity

**Figure 7:**
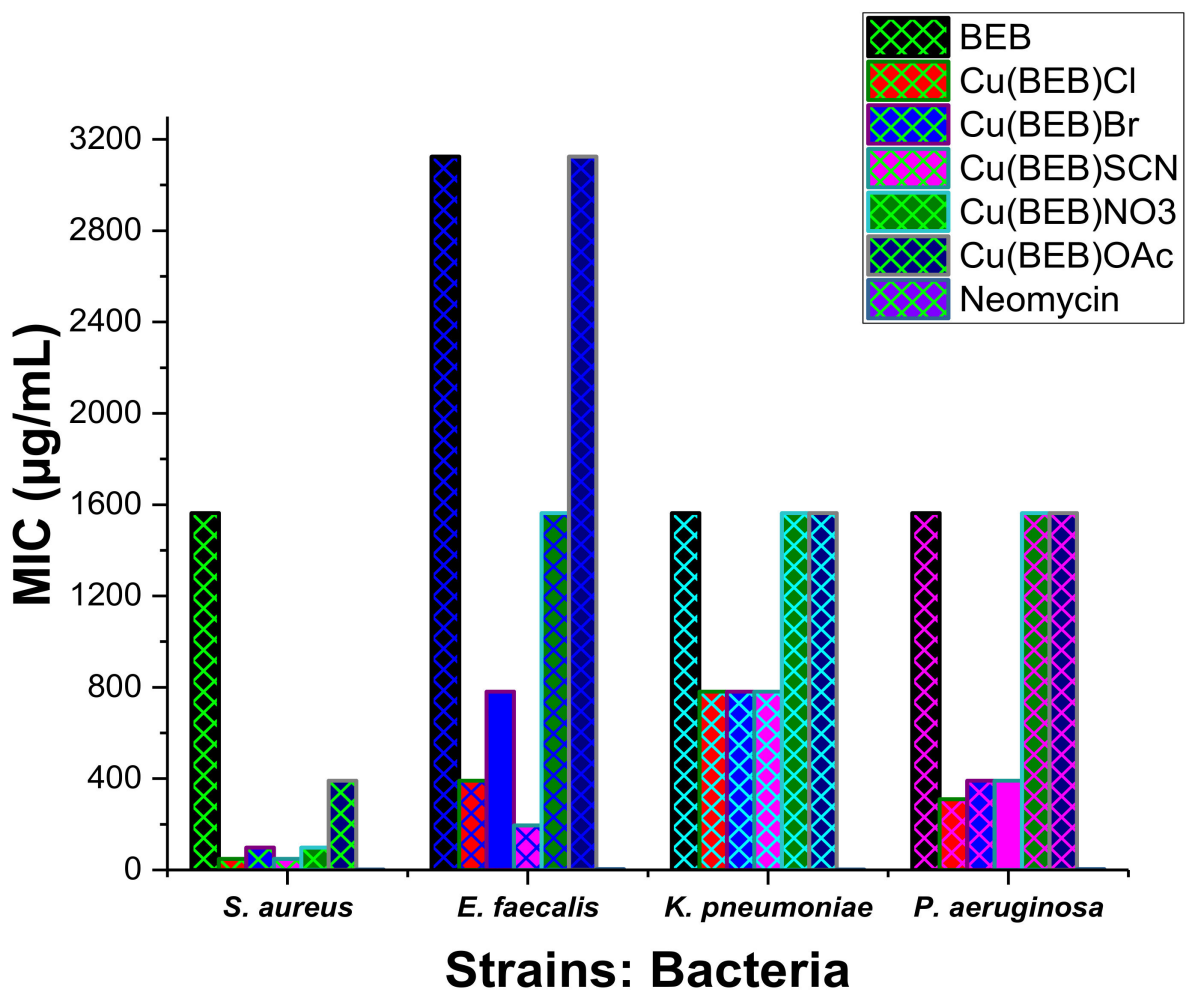
Antibacterial activity (MICs) of the Schiff base (BEB) and its Cu(II) complexes.

The antibacterial and antifungal activities of the Schiff base (BEB) and the corresponding heterocyclic copper complexes were investigated against bacterial strains: Gram-positive bacteria; *Staphylococcus aureus* (ATCC-25923) and *Enterococcus faecalis* (ATCC-29212), Gram-negative bacterial; *Klebsiella pneumoniae* (ATCC-13883) and *Pseudomonas aeruginosa* (ATCC-15442), and fungi; *Candida albicans* (ATCC-14053) and *Cryptococcus neoformans* (ATCC-14116). The antibiotic neomycin was used as a standard anti-bacterial control and amphotericin B as standard anti-fungal control in a *p*-iodonitrotetrazolium chloride (INT) colorimetric assay [30]. The compounds were tested at the final concentrations of 6250.00 to 48.83 μg/mL in DMSO as the solvent. The antimicrobial activities of the parent Schiff base (BEB) and its heterocyclic copper(II) complexes against bacterial and fungal strains are presented in Table 5. The activities of Cu(II) complexes bearing different anions showed activities that are higher than or comparable to the ligand, but lower in comparison to the standard antibiotic neomycin, and comparable antifungal activities to amphotericin B as a standard anti-fungi drug under the same study conditions (Figure 7). [Cu(BEB)Cl] and [Cu(BEB)SCN] exhibited good antibacterial activity, with MIC: 48.83 μg/mL against *S. aureus*; while [Cu(BEB)Br] and [Cu(BEB)NO_3_] showed an activity of 97.66 μg/mL against same bacterial strain (*S. aureus*). 

**Figure 8:**
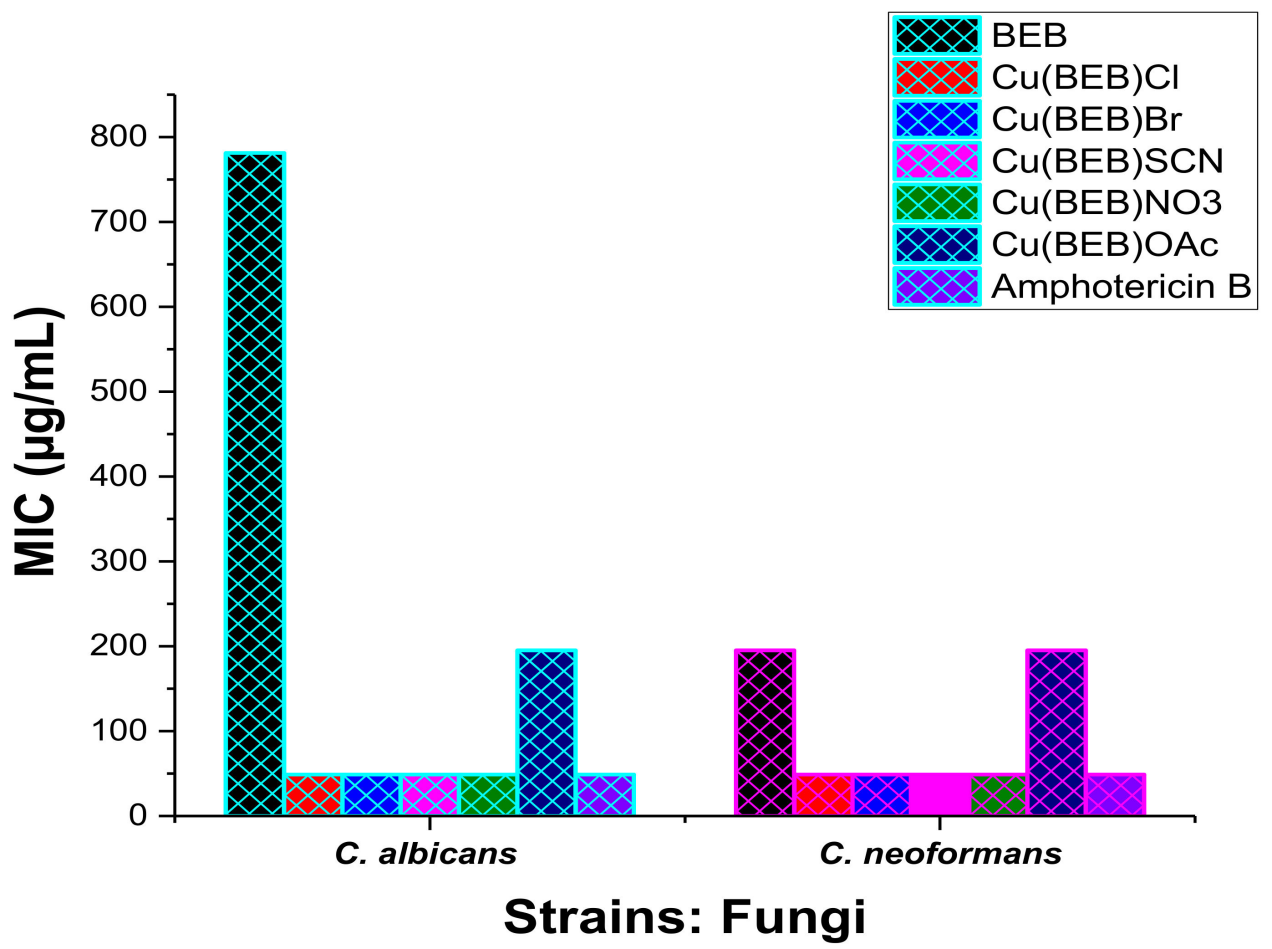
Antifungal activity (MICs) of the Schiff base (BEB) and its Cu(II) complexes.

All the complexes exhibited antifungal activity with MIC value of 48.83 μg/mL against *C. albicans* and *C. neoformans* (fungal strains), comparable to amphotericin B (an antifungal drug) except [Cu(BEB)OAc] with MIC value of 195.3 μg/mL (Table 5). The in vitro antifungal activity exhibited by the as-synthesized compounds (Figure 8) can be explained on the basis Overtone’s Concept and Tweedy’s Chelation Theory [10,16,23,26,31]. The minimum inhibitory concentration (MIC) displayed in Table 5 and Figure 7, describes the susceptibility of the selected microorganisms in the order; *S. aureus* (48.83 µg/mL) > *E. faecalis* (195.3 µg/mL) > *P. aeruginosa* (390.6 µg/mL) > *K. pneumoniae* (781.3 µg/mL) towards the [Cu(BEB)SCN]. The polarity of the copper ion is reduced to a greater extent due to the overlap of the ligand orbital upon complexation, and partial sharing of the positive charge of the Cu ions with donor groups. Chelation increases the delocalization of π-electrons over the whole ring, thereby enhancing the penetration of the complexes into lipid membranes [10,26,31]. It also increases the hydrophilic and lipophilic nature of the central metal atoms, subsequently leading to liposolubility and permeability through the lipid layer of cell membranes [23,29]. Formation of hydrogen-bonds through cell constituent active centers with the imino group (>CH=N–) leads to cell wall synthesis disruption which in-turn brings about the cytoplasmic membrane damage and cell death [10,19,29]. The respiratory processes of the cells might have been disturbed by the adsorption of the copper ions, thereby blocking their active sites, which restricts further growth of the organisms [9,15]. The particle size of the compounds also partly affects their antimicrobial activity, because nanosized particles exhibit increased antimicrobial activity [9,31]. The orders of the antimicrobial activity of the Cu(II) complexes of Schiff base ligand (BEB) is found to be:

For *Staphylococcus aureus* (ATCC-25923):

[Cu(BEB)Cl] = [Cu(BEB)SCN] > [Cu(BEB)Br] = [Cu(BEB)NO_3_] > [Cu(BEB)OAc].

For *Enterococcus faecalis* (ATCC-29212):

[Cu(BEB)SCN] > [Cu(BEB)Cl] > [Cu(BEB)Br] > [Cu(BEB)NO_3_] > [Cu(BEB)OAc].

For *Klebsiella pneumoniae* (ATCC-13883):

[Cu(BEB)Cl] = [Cu(BEB)Br] = [Cu(BEB)SCN] > [Cu(BEB)NO_3_] = [Cu(BEB)OAc].

For *Pseudomonas aeruginosa* (ATCC-15442):

[Cu(BEB)Cl] = [Cu(BEB)Br] = [Cu(BEB)SCN] > [Cu(BEB)NO_3_] = [Cu(BEB)OAc].

For *Candida albicans* (ATCC-14053):

[Cu(BEB)Cl] = [Cu(BEB)Br] = [Cu(BEB)SCN] = [Cu(BEB)NO_3_] > [Cu(BEB)OAc].

For *Cryptococcus neoformans* (ATCC-14116):

[Cu(BEB)Cl] = [Cu(BEB)Br] = [Cu(BEB)SCN] = [Cu(BEB)NO_3_] > [Cu(BEB)OAc].

## 3. Materials and Methods

### 3.1. Materials 

All the chemicals and solvents used were of analar or chemically pure grade and used without further purification during the syntheses processes. Ethylenediamine, benzaldehyde, NH_4_SCN, Cu(NO_3_)_2_∙3H_2_O were obtained from Merck (Johannesburg, South Africa), CuBr_2_ from Fluka (Buchs, Switzerland), while 2′,4′-dihydroxyacetophenone, CuCl_2_∙2H_2_O, (CH_3_COO)_2_Cu∙H_2_O, ascorbic acid, gallic acid were from Sigma-Aldrich (Johannesburg, South Africa). 1,1-Diphenyl-2-picrylhydrazyl (DPPH), 2,2′-azinobis-3-ethylbenzothiazoline-6-sulfonic acid (ABTS), rutin hydrate, and butylated hydroxytoluene (BHT), were purchased from Sigma Chemical Co. (St. Louis, MO, USA).

### 3.2. Physical Measurements

The analyses of carbon, hydrogen, and nitrogen were determined on a Perkin Elmer elemental analyzer (Waltham, MA, USA). Conductivity measurements were measured in freshly prepared 10^−3^ M DMF solutions at room temperature using a PC 7000 conductivity cell (EUTECH, Tuas, Singapore). The IR spectra were recorded on a Spectrum 2000 FT-IR spectrometer (Perkin Elmer) in the range 4000–400 cm^−1^. ^1^H-NMR spectra were recorded with an Advance DPX-600 MHz spectrophotometer (Bruker, Elisabethhof, The Netherlands) in *d*_6_-DMSO and reported relative to TMS as an internal standard. Powder X-ray diffraction (XRD) pattern was recorded on a Bruker AXS D8 Advance powder X-ray diffractometer (X-ray source: Cu, λ = 1.5406 Ǻ). Thermal Decomposition of the Complexes were recorded on Thermogravimetric analyzer: TGA 4000 System (Perkin Elmer, Waltham, MA, USA). Electronic spectra were recorded on a model T80+ UV-Vis spectrometer (PG Instruments Ltd., Leicestershire, UK) in the range 200–800 nm. Melting points were recorded with a SMP 10 Melting Point Apparatus (Stuart, Chelmsford, UK).

### 3.3. Synthesis of the Schiff Base Ligand: 4-[(1E)-N-{2-[(Z)-Benzylideneamino]ethyl}ethanimidoyl]benzene-1,3-diol (BEB)

The Schiff base ligand was prepared by a previously reported method [9]. Ethylenediamine (15 mmol) in ethanol (20 mL) was carefully added to solution of 2′,4′-dihydroxyacetophenone (15 mmol), in ethanol (30 mL) and stirred for 60 min at room temperature, followed by dropwise addition over 10–15 min of benzaldehyde (15 mmol) in ethanol (20 mL) at room temperature. The resulting mixture was refluxed with stirring for 3–4 h, and left to stir further for 22–24 h at room temperature to give the desired product as a crystalline solid after suction filtration, and washing with ethanol. The crude product was recrystallized from warm ethanol. The product was then dried in vacuum at 50 °C overnight with good yield (dark-yellow solid, 3.03 g, 71.5%).

### 3.4. General Procedure for the Synthesis of Cu(II) Complexes

The complexes were prepared by the addition of 1 mmol of copper salts [CuCl_2_∙2H_2_O (0.1705 g); CuBr_2_ (0.2234 g); Cu(NO_3_)_2_∙3H_2_O (0.2416 g); (CH_3_COO)_2_Cu∙H_2_O (0.1997 g)] dissolved in about 20 mL of ethanol, into a separate warmed ethanolic solution (20 mL) of BEB (1 mmol, 0.2823 g) in a 1:1 molar ratio. The colour of the solutions changed in a few minutes. The resulting mixture was refluxed for 2.5 h. The precipitated solids for the individual metal complex were filtered off from the reaction mixture, thoroughly rinsed with ethanol and dried over anhydrous calcium chloride (Scheme 1).

For the synthesis of Cu(BEB)-SCN 1 mmol of copper salt [CuCl_2_∙2H_2_O (0.1705 g)] dissolved in about 20 mL ethanol was added to a warmed ethanolic solution (20 mL) of BEB (1 mmol, 0.2823 g) in a 1:1 molar ratio. The colour of the solution changed in a few minutes. The resulting mixture was refluxed for 2 h. NH_4_SCN (1 mmol, in excess) was added and further refluxed for 1.5 h. The precipitated solid was filtered off, thoroughly washed with ethanol and dried over anhydrous calcium chloride (Scheme 1).

### 3.5. Biological Evaluations

#### 3.5.1. Antioxidant Assay

2,2-Diphenyl-1-picrylhydrazyl (DPPH) Radical Scavenging Activity

DPPH (1,1-diphenyl-2-picryl-hydrazyl) radical scavenging activity evaluation is a standard assay used in antioxidant activity studies. The antioxidant activity of the prepared Cu(II) complexes was studied spectrophotometrically by the DPPH method [24]. This method is a quick procedure for the screening of scavenging capacities of potential compounds [24]. The radical scavenging properties of the as-synthesized complexes and ligand with DPPH radical were assessed within different concentrations (100, 200, 300, 400, and 500 μg/mL) of the test compounds in DMF solutions (1 mL), added to 1.0 mL of 0.4 mM DPPH in methanol and thoroughly vortexed. The mixtures were allowed to incubate at room temperature in the dark for 30 min, after which the scavenging power of the test samples was measured with respect to decrease in the absorbance of DPPH at 517 nm. In this study, gallic acid, rutin and ascorbic acid (vitamin C) acted as standard drugs. Owing to the colour change, this signifies that the DPPH is scavenged by the antioxidant, through the donation of hydrogen to form stable DPPH molecule. It brings about an absorbance decreased. All tests analysis was performed in three replicates to obtain mean ± SD.

ABTS: 2,2′-Azino-bis(3-ethylbenzothiazoline-6-sulfonic Acid) Radical Scavenging Assay

ABTS scavenging ability of the Cu(II)-Schiff base complexes followed a previously described method [24]. Equal amounts of 7 mM ABTS solution and 2.4 mM potassium persulfate (K_2_S_2_O_8_) solution were prepared as the working solution and allowed to react further in the dark for 12 h at room temperature. An absorbance of 0.706 ± 0.001 units at 734 nm required for the analysis was obtained by diluting 1 mL ABTS^+^ solution. 1 mL of the test samples were mixed with 1 mL of the ABTS^+^ solution, followed by the absorbance reading spectrophotometrically at 734 nm. The samples’ ABTS scavenging potentials alongside standard drugs [gallic acid, rutin and butylated hydroxyl toluene (BHT)] was evaluated. Triplicate analyses were carried out.

The percentage inhibition of DPPH/ABTS radical scavenging activities were calculated following using the following equation [24]:

$$\left(\text{\%}\right)\;\mathrm{Inhibition}\;=\frac{Abs_{control}-\;Abs_{sample}}{Abs_{control}}\;\times \;100$$

where *Abs_control_* is the absorbance of the radical + solvent; *Abs_sample_* is the absorbance of radical + sample (test samples/standard).

#### 3.5.2. In Vitro Antimicrobial Studies

Micro-Dilution Bioassay

Microorganisms used in this study describe pathogenic species associated with different types of infections. The strains were maintained in the Biotechnology Laboratory at Vanderbijlpark, Vaal University of Technology and consisted of two Gram-positive (*Staphylococcus aureus* ATCC-25923 and *Enterococcus faecalis* ATCC-29212) and two Gram-negative (*Klebsiella pneumoniae* ATCC-13883 and *Pseudomonas aeruginosa* ATCC-15442). The cultures of bacteria were maintained on Mueller Hinton Agar (MHA) plates at 4 °C, while the fungi: *Candida albicans* (ATCC-14053) and *Cryptococcus neoformans* (ATCC-14116) were maintained on Yeast Malt (YM) broth plates at 4 °C throughout the study and used as stock cultures.

Determination of Minimum Inhibitory Concentration (MIC) (Antibacterial Activity)

Minimum inhibitory concentration (MIC) of the synthesized compounds (Schiff ligand and complexes) against the bacterial strains were determined by a rapid *p*-iodonitrotetrazolium chloride (INT) colorimetric assay [30]. Overnight cultures incubated at 37 °C in a water bath with an orbital shaker (Sallayicili: ST 30, Istanbul, Turkey) of Gram-positive: *Staphylococcus aureus* and *Enterococcus faecalis*; and Gram-negative: *Klebsiella pneumoniae* and *Pseudomonas aeruginosa* strains were diluted with sterile Mueller-Hinton (MH) broth to give a final inocula of approximately 10^6^ CFU/mL. Stock solutions of the synthesized compounds were dissolved in DMSO to a concentration of 25 mg/mL. One hundred (100) microliter of each test samples solution was serially diluted two-fold with sterile distilled water in a 96-well microtiter plate for each of the bacterial strains to a final concentration range of 6250.00 to 48.83 μg/mL. A 2-fold dilution of neomycin (Sigma-Aldrich, Darmstadt, Germany) (1 mg/mL) was used as positive control (antibiotic) against each bacterium to a final concentration range of 250.00 to 1.95 μg/mL, and dimethyl sulphoxide was used as a solvent control. One hundred microliters of the different bacterial culture were added to each well. The plates were covered with a sterile plate sealer and then incubated at 37 °C for 24 h in 100% relative humidity.

Bacterial growth was indicated by the addition of 40 μL of 0.2 mg/mL *p*-iodonitrotetrazolium chloride (INT) (Sigma-Aldrich) and further incubation at 37 °C for 24 h. Since the colourless tetrazolium salt is biologically reduced to a red product due to the presence of active organisms, the MIC values were recorded visually as the lowest concentration that led to growth inhibition. A reddish-pink colour indicated bacterial growth in the wells indicated bacterial growth.

Determination of Minimum Inhibitory Concentration (MIC) (Antifungal Activity)

A modified microdilution method for fungi as described by Masoko, Picard and Eloff [32] was used to determine the antifungal activity of the as-synthesized compounds against *Candida albicans* and *Cryptococcus neoformans*. An overnight fungal culture was prepared in yeast malt (YM) broth. Four hundred (400) microliters of this culture were added to 4 mL sterile saline, and absorbance read at 530 nm. The absorbance was then adjusted with sterile saline to give a 0.5 M McFarland standard solution. From this standardized fungal stock, a 1:1000 dilution with sterile YM broth was prepared to give a final inoculum of ≈0^6^ CFU/mL. Stock solutions of the synthesized compounds were dissolved in DMSO to a concentration of 25 mg/mL.

One hundred microliters of each solution were serially diluted two-fold with sterile water in a 96-well microtitre plate. A similar two-fold dilution of amphotericin B (Sigma-Aldrich) (2.5 mg/mL) was used as the positive control to a final concentration range of 625.00 to 4.88 μg/mL, while DMSO was used as a solvent control. One hundred microliters of the diluted fungal culture was added to each well. The plates were covered with a sterile plate sealer and incubated at 37 °C for 24 h. As an indicator of growth, 40 μL (0.2 mg/mL) INT was added and incubated for a further 24 h at 37 °C. MIC value was recorded as the lowest concentration that inhibited fungal growth after 48 h.

## 4. Conclusions

Five copper(II) complexes with a Schiff base ligand containing a N_2_O donor group [BEB] were synthesized and characterized by elemental analyses, IR, UV-Vis, NMR, thermal studies and XRD. The analytical data indicated the complexes as [Cu(BEB)Cl]·2H_2_O, [Cu(BEB)Br]·H_2_O, [Cu(BEB)SCN]·2H_2_O, [Cu(BEB)NO_3_]·3H_2_O, [Cu(BEB)CH_3_COO]·2H_2_O. Conductance measurements indicate that the complexes are non-electrolytes in solution. The spectral data show that BEB coordinates Cu(II) ion as a tridentate ligand through the nitrogen atom of the azomethine and oxygen atoms of the hydroxyl group of the 2′,4′-dihydroxyacetophenone after deprotonation. Square-planar structures were assigned to these complexes base on the elemental and spectral data. The thermal studies of these complexes provide information about the thermal stability, and the complexes gave a stable copper oxide residue above 450 °C. The XRD study suggests as-synthesized Cu(II) complexes possess a well-defined crystalline structure with average crystallite sizes of <60 nm. The as-synthesized compounds exhibited good antioxidant properties of scavenging free radicals. The results from DPPH and ABTS assays revealed that compounds are capable of donating electron or hydrogen atom and afterward react with free radicals or terminate chain reactions in a dose-dependent pattern. BEB and heterocyclic copper complexes along with conventional antibacterial drug: neomycin and fungicide amphotericin B were screened for antimicrobial activity against Gram (+ve) (*S. aureus* and *E. faecalis*), Gram (−ve) (*K. pneumoniae* and *P. aeruginosa*) bacteria, and fungi (*C. albicans* and *C. neoformans*). The mycological studies revealed that the compounds possess antibacterial and antifungal potential. 

## Figures and Tables

**Table 1:** Physicochemical and analytical data of [BEB] and its metal complexes.

Compounds	Empirical Formula	F. Wt (Gram)	Colour	Yield (%)	% Found (Calcd.)			Decomp. Temp, °C	Conductance (µS cm^−1^)
BEB	C_17_H_18_N_2_O_2_	282.34	Dark-yellow	71.50	72.17 (72.32)	6.31 (6.43)	9.86 (9.92)	233–234	-
[Cu(BEB)Cl]·2H_2_O	C_17_H_21_N_2_O_4_ClCu	416.36	Greyish	69.84	50.39 (49.04)	4.69 (5.08)	7.38 (6.73)	181–182	13.83
[Cu(BEB)Br]·H_2_O	C_17_H_19_N_2_O_3_BrCu	442.79	Dark-brow	75.49	47.87 (46.11)	5.13 (4.32)	6.82 (6.33)	175–176	13.15
[Cu(BEB)SCN]·2H_2_O	C_18_H_21_N_3_O_4_SCu	438.99	Dark-brown	60.72	48.21 (49.25)	5.29 (4.82)	10.29 (9.57)	178–179	15.09
[Cu(BEB)NO_3_]·3H_2_O	C_17_H_23_N_3_O_8_Cu	460.93	Dark-blackish	81.64	45.08 (44.30)	5.67 (5.03)	9.43 (9.12)	173–174	22.70
[Cu(BEB)CH_3_COO]·2H_2_O	C_19_H_24_N_2_O_6_Cu	439.95	Darkish-brown	65.09	50.94 (51.87)	5.01 (5.50)	6.89 (6.37)	171–172	10.71

**Table 2:** FT-IR Spectral data of the Schiff base [BEB] and its metal complexes.

Compound	ν(OH)/(H_2_O)	ν(CH_3_/CH_2_)	ν(SCN)	ν(C=N)	ν(C=C)	ν(NO_3_/OAc)	ν(C−O)	ν(M−N)	ν(M−O)
			asy&(sy)			asy&(sy)			
BEB	3404mb	3166w, 3057w		1613vs	1538m, 1436m		1266s		
[Cu(BEB)Cl]·2H_2_O	3384mb	3158w, 3067w		1588s	1538m, 1436m		1237s	594m	452m
[Cu(BEB)Br]·H_2_O	3392mb	3167w, 3064w		1589vs	1539m, 1435m		1237s	526m	455m
[Cu(BEB)SCN]·2H_2_O	3392mb	3157w, 3059w	2161m	1589vs	1539s, 1435w		1241s	567m	455m
			(2096m)						
[Cu(BEB)NO_3_]·3H_2_O	3378mb	3160w, 3062w		1590vs	1539m, 1436w	1417w	1240m	593m	457m
						(1297m)			
[Cu(BEB)CH_3_COO]·2H_2_O	3396mb	3162w, 3062w		1589s	1542w, 1435w	1538m	1240m	597m	462m
						(1340m)			

Abbreviations: s = strong; b = broad; v = very; m = medium; w = weak.

**Table 3:** Absorption data, band assignments of BEB ligand and its Cu(II) complexes.

Compounds		Absorption Transition, λ_max_ (nm, DMF)	Band Assignments
BEB	C_18_H_20_N_2_O_4_	277, 309, 381	π-π*, π-π*, n-π*
[Cu(BEB)Cl]·2H_2_O	C_18_H_20_N_2_O_5_Cu	325, 345, 375, 545	π-π*, L → M (LMCT), ^2^B_1g_ → ^2^A_1g_
[Cu(BEB)Br]·H_2_O	C_18_H_22_N_2_O_6_Cu	335, 395, 565	π-π*, L → M (LMCT), ^2^B_1g_ → ^2^A_1g_
[Cu(BEB)SCN]·2H_2_O	C_18_H_22_N_2_O_6_Cu	330, 380, 547	π-π*, L → M (LMCT), ^2^B_1g_ → ^2^A_1g_
[Cu(BEB)NO_3_]·3H_2_O	C_18_H_22_N_2_O_6_Cu	335, 395, 570	π-π*, L → M (LMCT), ^2^B_1g_ → ^2^A_1g_

**Table 4:** Activities of as-synthesized compounds and standard agents against DPPH* and ABTS* radicals.

Compounds	DPPH Radical Scavenging Activity		ABTS Radical Scavenging Activity	
	IC_50_ (µM)	R^2^	IC_50_ (µM)	R^2^
BEB	3.49 ± 1.57	0.913	2.77 ± 1.44	0.781
[Cu(BEB)Cl]	3.43 ± 0.82	0.965	3.02 ± 0.47	0.852
[Cu(BEB)Br]	3.40 ± 1.51	0.962	3.73 ± 0.77	0.739
[Cu(BEB)SCN]	2.60 ± 1.46	0.904	3.42 ± 0.41	0.917
[Cu(BEB)NO_3_]	2.01 ± 1.49	0.995	4.56 ± 1.99	0.766
[Cu(BEB)CH_3_COO]	2.88 ± 0.66	0.969	4.33 ± 1.62	0.778
Vit. C *	1.74 ± 1.19	0.976	-	-
Rutin *	2.49 ± 1.27	0.835	2.86 ± 0.92	0.931
Gallic acid *	1.44 ± 1.11	0.890	1.22 ± 1.08	0.949
BHT *	-	-	2.31 ± 1.30	0.971

(n = 3, X ± SEM), IC_50_—Inhibitory concentration; shows the percent inhibition of the examined compound at 50%, R^2^ = correlation coefficient. * Standards.

**Table 5:** Schiff base ligand [BEB] and its Cu(II) complexes minimum inhibitory concentration (MIC) (µg/mL).

Compounds	Gram (+) bacteria		Gram (−) bacteria		Fungi	
	*S. aureus*	*E. faecalis*	*K. pneumoniae*	*P. aeruginosa*	*C. albicans*	*C. neoformans*
BEB	1563	3125	1563	1563	781.3	195.3
[Cu(BEB)Cl]	48.83	390.6	781.3	390.6	48.83	48.83
[Cu(BEB)Br]	97.66	781.3	781.3	390.6	48.83	48.83
[Cu(BEB)SCN]	48.83	195.3	781.3	390.6	48.83	48.83
[Co(BEB)NO_3_]	97.66	1563	1563	1563	48.83	48.83
[Cu(BEB)CH_3_COO]	390.6	3125	1563	1563	195.3	195.3
Neomycin ^a^	1.953	3.906	1.953	3.906	-	-
Amphotericin B ^a^	-	-	-	-	48.83	48.83

^a^ Standards.
